# Current applications and prospects of nanoparticles for antifungal drug delivery

**DOI:** 10.17179/excli2020-3068

**Published:** 2021-03-08

**Authors:** Sanam Nami, Ali Aghebati-Maleki, Leili Aghebati-Maleki

**Affiliations:** 1 Department of Parasitology and Mycology, Faculty of Medicine, Tabriz University of Medical Sciences, Tabriz, Iran; 2 Student Research Committee, Tabriz University of Medical Sciences, Tabriz, Iran; 3 Immunology Research Center, Tabriz University of Medical Sciences, Tabriz, Iran; 4 Department of Immunology, Faculty of Medicine, Tabriz University of Medical Sciences, Tabriz, Iran

**Keywords:** nanoparticles, drug delivery, antifungal drug

## Abstract

Currently, the significance of fungi as human pathogens is not medically concealed in the world. Consequently, suitable recognition and treatment of such infections are of great importance and necessitate the need for comprehensive information in this regard. The introduction of new antifungals and their use today, especially in the last two decades, have revolutionized the treatment of fungal infections. On the other hand, increasing drug resistance in the world has overshadowed such developments. The use of NPs results in the treatment of fungal infections and owing to their specific properties, these particles, unlike the pure antibiotics, can exert a greater inhibitory power although with less concentration compared with conventional drugs. Important reasons that have led to the use of antifungal drugs in delivery systems include reduced drug efficacy, limited penetration through tissue, poor aqueous solubility, decreased bioavailability, and poor drug pharmacokinetics. It is therefore hoped that unfavorable properties of antifungal drugs be mitigated via their incorporation into different types of NPs. This review summarizes the different types of NPs as delivery systems of antifungal as well as their advantages over pure drugs.

## Introduction

Nowadays, the significance of fungi as human pathogens is medically transparent in the world. Fungi that were not previously infectious in humans are nowadays among the opportunistic pathogens which are increasing in number. Thanks to their ability to adapt to a variety of environmental conditions, these fungi can easily endanger the lives of immune-deficient patients and are now one of the leading causes of death for these patients. Therefore, timely recognition and treatment of such infections are of great importance and necessitate the need for comprehensive information in this regard. Studies show that more than 300 million people suffer from serious fungal infections, accounting for about 1.4 million deaths annually (Brown et al., 2012[18]). The introduction of new antifungals and their use today, especially in the last two decades, have revolutionized the treatment of fungal infections. On the other hand, unfortunately, increasing drug resistance in the world has overshadowed such developments. In developed countries, antiretroviral therapy (ART) has led to a marked decrease in the incidence of fungal infections among people with human immunodeficiency viruses (HIV), but lack of access to such drugs in the developing countries has brought about a noticeable surge in the incidence of these infections. On the other hand, the use of invasive therapies in intensive care units, use of immunosuppressive drugs after organ transplantation, treatment of malignancies, increased use of antifungal prophylaxis with azole derivatives, as well as mounting use of amphotericin B for the empirical therapy have led to significant changes in terms of heightened incidences of fungal infections with new patterns as well as the development of drug resistance, especially in opportunistic fungi (Fisher et al., 2018[40]). Therefore, researchers spare no effort to minimize drug resistance and toxicity using efficient methods.

Today, nanoparticles (NPs) have been highly appreciated for their wide range of applications to various biological, pharmacy and medical fields. Structurally considered, their size barely exceeds the range of 100 nm. A broad spectrum of drugs, such as small hydrophobic and hydrophilic drugs, vaccines and biological molecules can be controlled by these NPs (Gupta and Xie 2018[48]). NPs are extensively used in tissue engineering scaffolds, targeted drug delivery, and also the diagnosis of diseases (Mura et al., 2013[77]). NPs have been widely exploited in forms of nanoliposomes, carbon nanotubes, nanofibers, nanocapsules as drug carriers, and cellular scaffolds. The primary purpose of producing NPs as a drug delivery system is to control particle size, surface properties, and the effective delivery of a specific drug at a particular time and place for exerting the maximum effect (Mu and Feng, 2003[75]). The NPs used for drug delivery not only should possess both biocompatibility and biodegradability properties but also their timely release, optimum mechanical properties and ease of production need to be taken into account. NPs get trapped in body through the circulatory system or phagocytosis which can be tracked through surface modification and thus, be saved in the circulation system (Mahapatro and Singh, 2011[67]).

**Figure 1:**
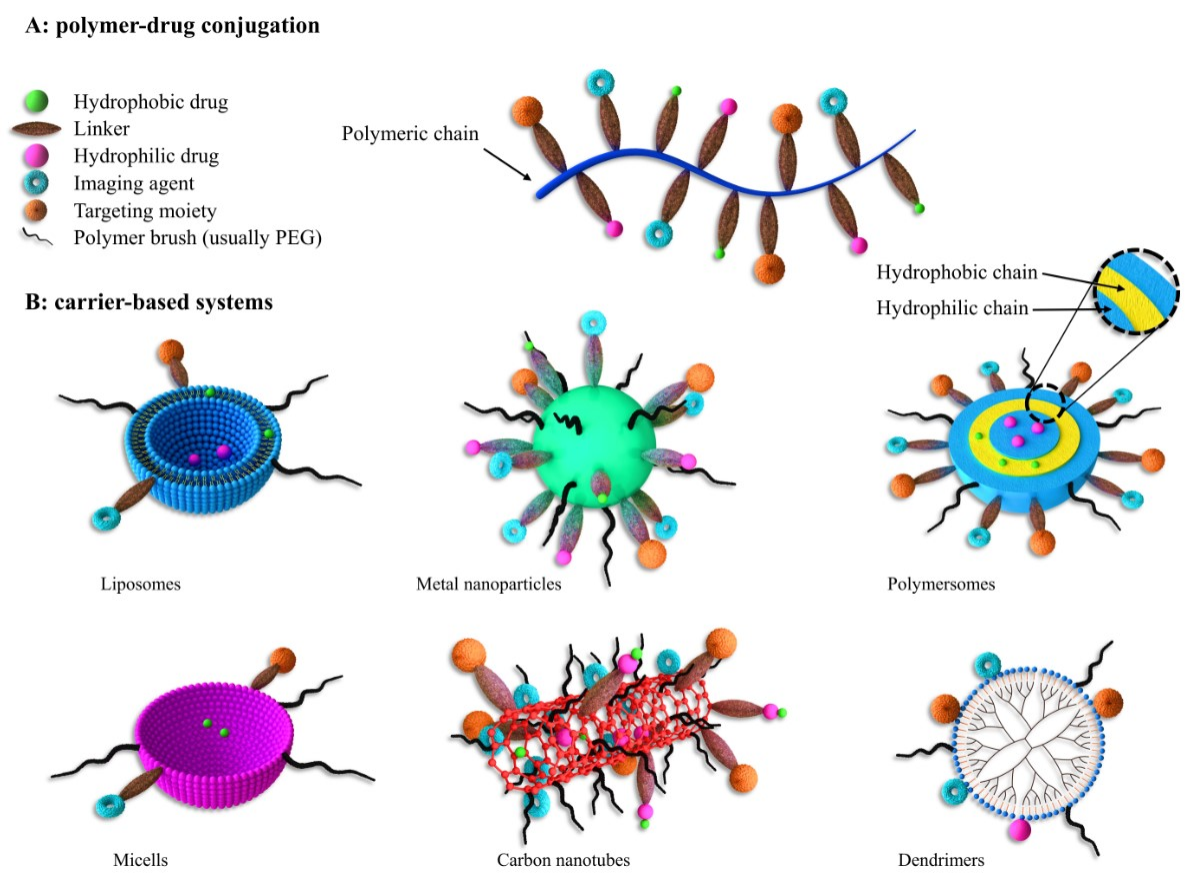
Different types of nano carriers for drug delivery

NPs can be categorized in a variety of ways in terms of their size, shape and constituting materials (Figure 1(Fig. 1)). Even the preparation methods of NPs can lead to the creation of a variety of NPs, with each of them having different loading capacity, delivery, and shelf-life (Hu et al., 2010[54]). They are divided into dendrimers, nanospheres, nanocapsules, liposomes, micelles, polymersomes, fullerenes and nanotubes according to their appearance. Other studies have classified them into organic and non-organic groups. The organic molecules are the main components of the NPs within the organic category whereas in the mineral category, metals (iron, gold, etc.) and other mineral elements play a pivotal role in the structure of the NPs (Erdoğar et al., 2019[36]). Liposomes, dendrimers, carbon nanotubes, solid lipid NPs, and polymers belong to the organic particle category while mineral NPs contain a central core made of mineral or metal elements covered by a coating of organic materials. The cores depict fluorescence, magnetic, and electrical properties (Frank et al., 2015[41]). In another classification, NPs are made of macromolecular or polymeric materials, natural or synthetic, and are classified into two types of nanosphere and nanocapsule based on their production methods. Nanocapsules are sac-like structures in which the drug is placed in a central chamber surrounded by a polymeric layer. Nanosphere is a matrix system within which the drug and polymer are either homogeneously dispersed or absorbed on the surface. Polymers, being applied as NPs, are accompanied by the drugs with specific therapeutic effects for specific diseases such as cancers. These NPs are bonded to nanocomposites in two ways: 1. The drugs are encapsulated in nanocarriers; 2. The drugs are conjugated over the nanoparticle surface (Moritz and Geszke-Moritz, 2015[74]). So far, various polymers including polyamide, polyamino acid, poly (alkyl α-cyanocrylates), polyester, polyorthoester, polyurethane, polyacrylamide, and polycaprolactone have been used as drug carriers. Amongst them, aliphatic polyester thermoplastics such as polylactic acid (PLA), polyglycolic acid (PGA), and poly lactic-co-glycolic acid (PLGA) as the copolymer of these two have been widely used compared to others due to their super biocompatibility and biodegradability properties (Cheung et al., 2015[26]). Langer and Folkman were the pioneers to reveal the controlled release of macromolecules through polymers which led to the advancement of the drug release system in anti-angiogenics for cancers (Langer and Folkman, 1976[61]). Polymer-based NPs are a proper tool for biomolecules, drugs, genes and vaccines guidance. The solubility and shelf life of the drugs can be honed by being encapsulated within NPs. By doing this, the main objectives are targeted drug transfer with a determined concentration within the range of treatment and ultimately the patient's satisfaction (Dinarvand et al., 2011[34]). Polymer carriers have received a great deal of attention in the last few decades thanks to their multiple functions and their potential to be functionalized. In general, polymer NPs are minute colloid systems in which the drug is physically dispersed or dissolved or chemically bonded to their main polymer chains. Among the advantages of utilizing polymer NPs as a pharmaceutical nanocarrier is the enhanced drug solubility and stability. Hence, polymer NPs are considered to be the most widely administered drug release systems (Hickey et al., 2015[52]). To date, several polymers have been investigated based on their characteristics, some of which are as follows: 

Chitosan, a carrier of NPs, is a plentiful natural polysaccharide known for its beneficial properties. Compared to the synthetic polymers, chitosan obtained by chitin acetylation is suitable for the formation of nanogels due to its biocompatibility, better biodegradability, and stability as well as low toxicity (Onyebuchi and Kavaz, 2019[83]; Saito et al., 2019[97]). However, the low solubility of chitosan in the alkaline and neutral medium due to the large number of amino groups in its chain on the one hand, and the formation of strong hydrogen bonds and stable crystal structure on the other hand limits its use in pharmaceutical and biomedical applications (Rodrigues et al., 2012[94]; Bruinsmann et al., 2019[19]). As a result, some different chitosan amendments have been implemented, but these may include toxicity issues. To overcome such restrictions, polyethylene glycol methyl ether is an excellent hydrophilic polymer whose bond with chitosan chain not only improves the compatibility of chitosan but also prevents protein absorption and escape from the reticuloendothelial system (Szymańska et al., 2016[110]; Antoniraj et al., 2018[9]). 

PLGA is one of the most successful polymers used for drug release applications, several outstanding benefits of which include biocompatibility, drug compatibility, mechanical properties, easy production process; besides, its hydrolysis results in the formation of monomers of lactic acid and glycolic acid metabolites. Since these two monomers are androgens and are easily metabolized by body through the Krebs cycle or TCA cycle, PLGA induces poor systemic toxicity (Richard and Margaritis, 2001[93]; Yang, 2019[121]). PLGA NPs protect the drug against degradation and improve its stability (Le Garrec et al., 2004[62]; Tian et al., 2017[113]; Yang, 2019[121]). These systems also have some downsides; namely, rather low drug loading for many drugs, costly production and the issues pertaining to expanding the scale of use (Moritz and Geszke-Moritz, 2015[74]). Relatively low drug loading is probably the major obstacle to the use of drug-loaded PLGA NPs in clinical trials. As a result, further progress is needed to transform the concept of drug-loaded PLGA NPs into a realistic action plan as a new generation of drug delivery systems with the primary goal of enhancing the therapeutic effects and minimizing side effects (Le Garrec et al., 2004[62]). 

Proteins are a bountiful source of natural materials for making nanoparticle systems. Various proteins including gelatin, albumin, and curcumin are widely applied for drug delivery (Verma et al., 2018[119]). The use of protein NPs for treatment is yielding astonishing results and promises greater efficacy in the future. With respect to functional comparisons and therapeutic efficacy, proteins and other existing delivery systems still suffer from some defects, necessitating further investigations in this area (Tosi et al., 2013[114]). 

Many studies are being conducted worldwide today concentrating on the improvement of therapeutic methods for corneal fungal infections and invasive mycoses in particular, due to the poor bioavailability of antifungal drugs that arise from various protective mechanisms. Numerous *in vivo* investigations indicated that the accumulation of drug-loaded nanoparticles in lungs was significantly higher than that of a pure drug; in addition to this, the prolonged retention of particles in lungs was observed (Balabathula et al., 2020[14]; Faustino and Pinheiro, 2020[39]). Sometimes oral therapy is not adequate especially for topical fungal infections; thus, topical application of this drug formulation can yield better results such as improved skin penetration due to enhanced contact between antifungal and skin. One of the preferred mechanisms in advancing drugs for better penetration is the use of these nanosystems (Dhamoon et al., 2019)[33]. So in general, due to the limited antifungals, we are facing a number of problems such as prolonged treatment times, high costs, complex route of administration, side effects, drug resistance, toxicity, and drug interactions (Souza and Amaral, 2017[106]). The benefits of using these NPs in combination with antifungals entail increased solubility, improved permeability, bioavailability, enhanced storage stability, prolonged half-life, reduced therapeutic cost, and adequate dose of drugs (Faustino and Pinheiro, 2020[39]). In this review, we will summarize the major findings related to nanoparticles and some of the strategies using nanotechnology to improve the conventional therapy.

## Types of Smart Drug Delivery

Recent clinical and pre-clinical studies have revealed that targeted drug delivery systems are a great way to treat various life-threatening diseases. Smart drug delivery is divided into the following categories: 

### Nano-scale carriers

Drug delivery using nanocarriers is absolutely desirable due to their very small diameters (10 to 1000 nm) which greatly contributes to improved encapsulated drug treatment efficacy (Kumar Giri et al., 2016[59]). In recent years, many nanometer structures have been investigated and developed for drug delivery purposes including nanoliposome, polymer NPs, solid lipid NPs, and dendrimers. 

#### Nanoliposome

**Figure 2:**
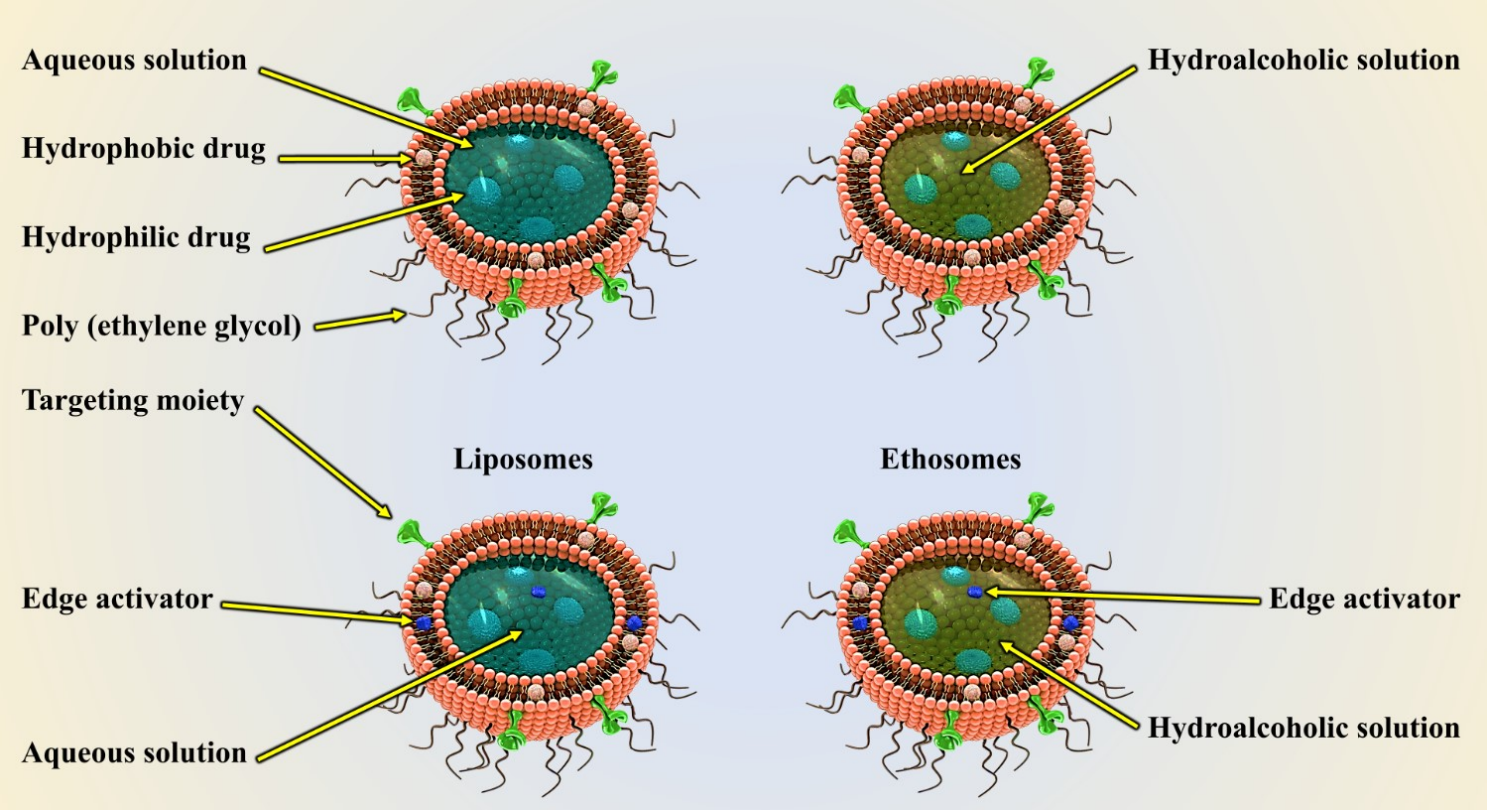
Liposomes are bilayered vesicles having an aqueous core and one or more concentric phospholipid membranes. This structure allows liposomes to act as effective delivery systems for both hydrophilic and hydrophobic drugs.

Nanoliposomes are self-forming nanostructures that come together in an aqueous solution of lipid molecules. Phospholipid lipophilic molecules are grouped in such a way that their hydrophobic groups are directed inward whereas their hydrophilic groups are aligned in the outer layer of the sphere, thus forming a spherical bilayer membrane. This mode of orientation allows the loading of hydrophilic drugs in the nucleus, and hydrophobic agents in the liposomes shell (Figure 2(Fig. 2)) (Gunasekaran et al., 2014[46]). Today, these nanostructures are used as a drug or gene carriers, and for modeling purposes of cell membranes, both in animals and in humans. The ability of these nanostructures in encapsulating large quantities of drugs, minimizing unwanted side effects, and having high efficacy and low toxicity has attracted researchers' interest. Also, other benefits of nanoliposomes are the ease of production in industrial volumes, excellent fabrication quality, variety in particle size, chemical composition, and electrical charge. Liposomes have a broad particle size range, spanning from micron-sized macroliposomes to nano-sized liposomes. Nanoliposomes (liposomes of less than 200 nm) are frequently used in the pharmaceutical industry. These liposomes can easily pass through a variety of obstacles they face such as blood barriers (Moghimipour et al., 2012[70]).

#### Polymer nanoparticles

Polymeric NPs as pharmaceutical carriers are made up of both biodegradable polymers and non-biodegradable types. In recent years, their biodegradable formulation has attracted considerable attention as potentially suitable drug delivery systems due to their ability to release drugs modestly, to load large amounts of pharmaceuticals and prevent drug degradation (Faraji and Wipf, 2009[38]). In this system, the drugs are either trapped or bound by covalent bonding to the polymer matrix. Besides, polymer NPs are also used to improve the surface quality that can in turn, enhance drug absorption efficiency. Polyethylene glycols have been widely used in polymer NPs to hone biodegradation (Cho et al., 2008[28]; Pérez-Herrero and Fernández-Medarde, 2015[87]). In general, polymers used in the preparation of these structures fall into two categories: natural polymers such as chitosan, albumin and heparin, and synthetic polymers such as (HPMA) N- (2-hydroxypropyl) methacrylamide PLA, PGA, and PLGA. However, given the importance of biodegradability and safety, natural polymers are preferably used to deliver a wide range of drugs from macromolecules to small molecules. 

#### Solid lipid NPs

Solid lipid NPs (SLN) are colloidal structures that can be prepared by emulsification and by means of mechanical forces such as ultrasound and homogenizer, they get reduced to below micrometers in size. These systems are formed by replacing the w/o emulsion oil phase with a solid oil or a mixture of solid oils; that is, a mixture of lipid matrix particles that are solid at room temperature and body. SLN consist of 0.1 to 30 % solid fat dispersed in the liquid phase and if necessary, 0.5 to 5 percent of the surfactant is also used in their preparation. The average SLN particles range from 40 to 1000 nm. Studies have shown that the physicochemical and stability properties of drugs loaded in the SLN depend on the properties of the drugs and the components used. These structures are capable of carrying drugs and active substances in their lipid portion, thereby protecting the target material from environmental damages. As a result, this spectrum of NPs can be used to deliver drugs and prolong their effectiveness (Li et al., 2017[63]; Rodriguez-Torres et al., 2020[95]). Drug delivery by SLN depends on various factors such as the way the samples are administered, the type of lipid and active ingredient used, as well as the type of interaction of the body with the particles (Qu et al., 2016[89]). The most important enzyme that affects these structures in the body is lipase. The rate of degradation of different lipids varies with this enzyme. For example, the longer the length of the lipid chain, the slower the effect of the enzyme on its degradation. In the presence of some emulsifiers, the degradation rate also decreases and the emulsifier acts as a lipid shield (Cunha et al., 2017[30]).

#### Dendrimers

Dendrimers are a family of three-dimensional, nano-sized polymers characterized by a compact spherical structure in solution. Although the origin of dendrimers is linear polymers and then branched polymers, the remarkable structural properties of high branched dendrimers and macromolecules are quite different from those of traditional polymers (Bugno et al., 2015[20]). Despite the use of polymers in drug delivery systems, dendrimers are more beneficial than them. They have finite polydispersity and nanometer dimensions that facilitate passing through biological barriers. Dendrimers can encapsulate guest molecules by receptors at their surface or within the cavities between branches (Fréchet, 2003[42]). Unlike linear polymers, dendrimers are macromolecules that branch out of a single nucleus, with all the branches eventually reaching a central nucleus. In making dendrimers, their molecular size and mass can be precisely controlled. The presence of a large number of terminal branches increases the solubility and reactivity of the dendrimers. The solubility of dendrimers is strongly influenced by the nature of the surface groups. For example, the presence of hydrophilic groups makes the dendrimers soluble in polar solvents whereas the hydrophobic end groups make the dendrimers more soluble in nonpolar solvents. The importance of dendrimers is highlighted since the therapeutic effect of any drug depends on its optimal solubility in the aquatic environment. There are numerous substances with strong therapeutic properties but are not used for therapeutic purposes due to their insolubility. Water-soluble dendrimers can bind to hydrophobic molecules with antifungal or antibacterial properties. The release of the bound drug is plausible upon contact with the target organisms and thus, these complexes are considered as drug delivery systems (Bugno et al., 2015[21]). 

#### Nanocapsules

Nanocapsules consist of a thin membrane surrounding a core (Liquid, solid) with their size ranging from 10 nm to 1000 nm. Nanocapsules are submicroscopic colloidal drug carrier systems composed of an oily or an aqueous core surrounded by a thin polymer membrane. The membrane may be composed of natural or synthetic polymers (Mora-Huertas et al., 2010[73]). Two technologies can be used to obtain such nanocapsules: the interfacial polymerization of a monomer and the interfacial nano deposition of a preformed polymer. The nanometric size of nanocapsule can be interesting for many applications like cosmetics, perfumes or pharmaceutical industries. The nanocapsule is used in various fields: for drug delivery in case of tumors, or as a nanocapsule bandage to fight infection, as a liposomal nanocapsule in food science and agriculture, in delivering radio therapy and as a self-healing material. Nanocapsules can be used as smart drugs that have specific chemical receptors and only bind to specific cells. It is this receptor that makes the drug 'smart, allowing it to target cancer or disease. The advantages of nano-encapsulation technologies for pharmaceutical applications include: higher dose loading with smaller dose volumes, longer site-specific dose retention, more rapid absorption of active drug substances, increased bioavailability of the drug, higher safety and efficacy, and improved patient compliance (Radhika and Sivakumar, 2011[90]).

### Targeted pro-drug 

Pro-drugs are inactive biological compounds that become active after encountering a specific physiological barrier (Hoste et al., 2004[53]). The pro-drugs are designed to improve parameters such as undesired side effects, solubility, stability, biodegradability, toxicity and systemic metabolism. In recent years, a group of pro-drugs called targeted precursors have received much attention (Greco and Vicent, 2008[45]). In general, a targeted drug contains a parent drug or its derivatives, a bond breakable by a chemical or enzymatic activity such as amide and ester, an enzymatically or chemically breakable spacer, and a transient target structure (Mahato et al., 2011[68]). Precise selection of the breakable bond and the target structure of the transition play a critical role in the construction of the targeted drug. The breakable bonds commonly used in the manufacture of pro-drugs include amides, esters, disulfide bonds, and phosphate esters, among which ester and amide are more widely used. The ester bond is usually broken by the esterase enzyme which is highly dispersed in the body. This stability problem can be tackled by replacing carboxyl esters or phosphate esters with carbamate esters (D'Souza and Topp, 2004[31]). Other breakable bonds, such as amines bond / oxime bond along with thioether non-breakable bond are used in the manufacture of targeted precursors. The choice of the linker depends on the use of the pro-drug. Disulfide bonds, for example, are commonly used to target cancerous tissues because the glutathione in these types of tissues breaks the bond. Similarly, hydrazone acid-sensitive bonds release drugs into endosomal structures at low pH (D'Souza and Topp, 2004[31]; Tai et al., 2011[111]).

## NPs for Antifungal Drug Delivery

**Figure 3:**
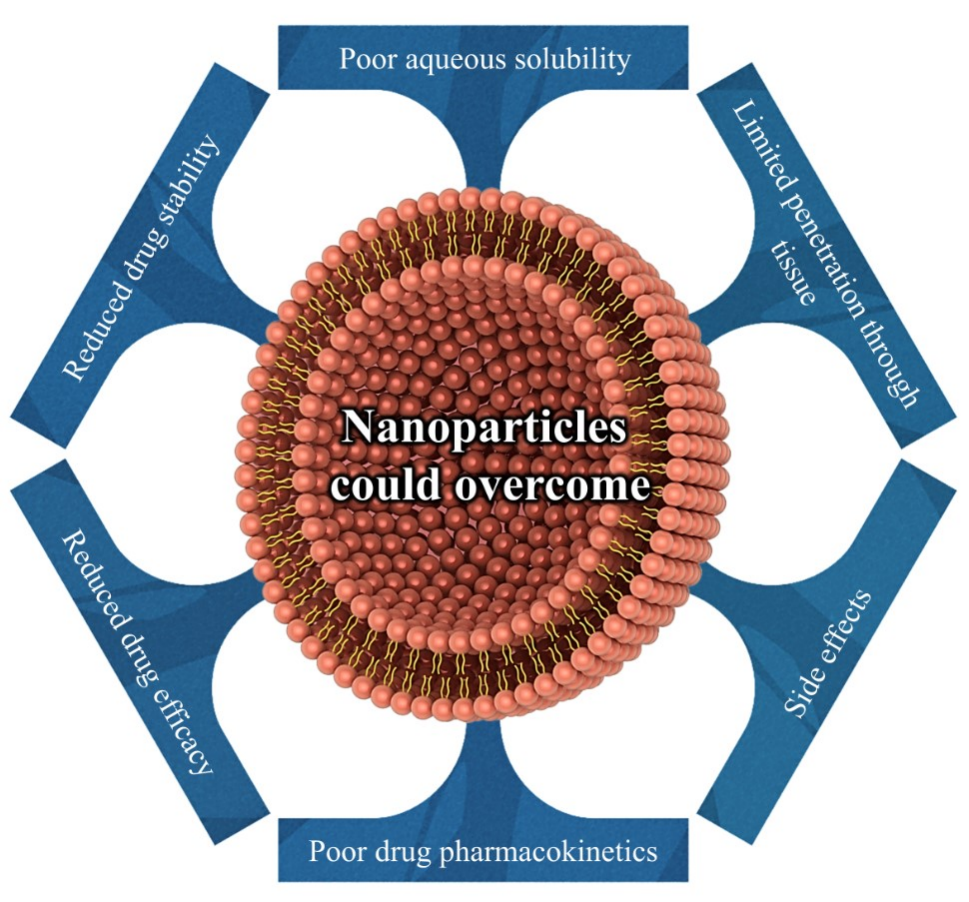
NPs can overcome many of the unfavorable drug properties through virtue of their versatility, multifunctionality and wide range of properties.

The main classes of antifungal medications used in mycoses treatment include polyenes (amphotericin B (deoxycholate, and lipid-based), hamycin, natamycin, nystatin), azoles (imidiazoles (clotrimazole, econazole, ketoconazole, miconazole, …), triazoles (fluconazole, itraconazole, isavuconazole, voriconazole, ravuconazole, posaconazole, …), and thiazole (abafungin)), pyrimidine analogue (flucytosine), echinocandins (caspofungin, anidulafungin, micafungin, cilofungin, and biafungin), squalene monooxygenase (allylamines (naftifine, and terbinafine), benzylamine (butenafine)), mitotic inhibitor (griseofulvin) (Campoy and Adrio 2017[23]; Nami et al., 2019[79]). Given all the advances in the optimal development of such classes of antifungal medicine, the use of NPs drug delivery systems has been proposed for their exceptional efficiency as well as their maximized activity. The use of NPs results in the treatment of fungal infections and owing to their specific properties, these particles, unlike the pure antibiotics, can exert a greater inhibitory power although with less concentration compared with drugs. Important and prominent reasons that have led to the use of antifungal drugs in delivery systems include reduced drug efficacy, limited penetration through tissue, poor aqueous solubility, decreased bioavailability, reduced drug stability, side effects, and poor drug pharmacokinetics (Soliman, 2017[102]; Hassanpour et al., 2020[50]). It is therefore hoped that encapsulating drugs in NPs (≤ 100 nm) can partially overcome such problems of antifungals (Figure 3(Fig. 3)). In the following, we will discuss the different types of NPs as delivery systems of antifungal as well as their advantages over pure drugs.

### Solid lipid nanoparticle

Today, the delivery of antifungal drugs to the target skin region is one of the major problems when using medications systemically. The use of systemic drugs in the treatment of cutaneous diseases, in addition to having various side effects, can cause poor drug availability at the infection site. Therefore, one of the main goals of accessing an effective novel drug delivery route is to increase drug localization in the targeted organ. In recent years, SLNs have drawn more attention in topical applications due to their chemical affinity for the skin. Several prominent advantages that make SLNs apt for drug delivery systems include their nanometer range, sustained release, biocompatibility, and reduced side effects, with lipid matrix being the leading cause of a reduction in the risk of toxicity (Pople and Singh, 2006[88]; Trombino et al., 2016[115]). Studies have shown that the physicochemical and stability properties of drugs loaded in the SLNs depend on the properties of the drugs and the components used. These structures are capable of carrying drugs and active substances in their lipid portion, thereby protecting the target material from environmental damage. As a result, this spectrum of NPs can be used to deliver drugs and prolong their effectiveness (Rodriguez-Torres et al., 2020[95]). SLNs are also used in the delivery of antifungal drugs for topical applications such as amphotericin B (AmB), azoles, griseofulvin (GF), etc, some of which will be discussed later on. In a study by Butani et al., in 2016 on AmB, it was suggested that SLNs were effective for the topical delivery of AmB. He noted impressive advantages of using AmB loaded SLNs compared to drugs only, including higher skin deposition, lower skin irritation, and better antifungal activity. The researcher used several SLNs formulations of AmB in his studies, asserting that SLN5 (lipid ratio 1:10 and Pluronic F 127 0.25 % as a surfactant) have the best formulation. He also showed that the SLN5 formulation on the *Trichophyton rubrum* fungus has a higher zone of inhibition compared to the pure drug (Butani et al., 2016[22]). Another antifungal cream in the treatment of cutaneous disease is clotrimazole (CLT) and in several studies, SLNs were employed for the encapsulation of CLT. In a study by Souto et al., in 2004 on azole drug, CLT-loaded SLN was developed by hot high-pressure homogenization to demonstrate the physical stability of these lipid particles in their study (Souto et al., 2004[104]). Cassano et al. (2016[24]) investigated the vaginal infections sustained by *Candida albicans*. The researchers used SLNs based on polyoxyethylene-40 stearate for the administration of CLT, all yielding satisfactory results in the treatment of vulvovaginal candidiasis infection. Nowadays, recurrent vulvovaginal candidiasis (≥ 4 episodes within 1 year) is a common cause of morbidity affecting millions of women worldwide; however, it is hoped that by further studies on this new formulation and clinical trials, this drug can be used to treat the so-called patients (Cassano et al., 2016[24]). Another topical medication is econazole (ECN), whose poor water solubility has limited its bioavailability and antifungal effects. To this end, Sanna et al. (2007[100]) worked on the econazole nitrate drug using the o/w high-shear homogenization technique, by which SLN was designed for the topical administration of ECN to improve ECN penetration through skin. In this *in vivo* study, they showed that SLN promoted an immediate penetration of ECN through the stratum corneum compared to conventional gel (Sanna et al., 2007[100]). A study by Bhalekar in 2009 on miconazole nitrate (MN), who produced MN loaded SLN by hot homogenization, revealed that MN-SLN formulations increased the accumulation of MN in the skin as opposed to the conventional gel and improved skin targeting effect (Bhalekar et al., 2009[17]). In 2010, Jain et al., conducted a similar study with MN-loaded SLNs, produced by a modified solvent injection method. The researchers used an animal model of rats infected with *Candida* species and examined them for cutaneous candidiasis. They reported that MN-loaded SLN-bearing hydrogel was more effective than the free drug in the treatment of candidiasis (Jain et al., 2010[56]). Also, Aljaeid and Hosny developed MN-SLNs produced by hot homogenization /ultrasonication, to overcome the problem of poor aqueous solubility of this drug. They investigated antifungal activity against *Candida albicans* during the *in vitro* tests and used rabbits *in vivo* to ultimately observe the increased oral bioavailability of MN-SLN compared to the commercially available capsules (Aljaeid and Hosny, 2016[7]). Other studies on MN include the 2017 study by Kenechukwu et al., by which solidified reverse micellar solution-based mucoadhesive nano lipid gels encapsulating SLNs were developed and evaluated for improved localized oromucosal delivery of MN for effective treatment of oropharyngeal candidiasis (Kenechukwu et al., 2017[57]). One of the triazoles, widely used in the treatment of fungal infections today, is a case of itraconazole (ITZ) developed by Mohanty et al., in 2015 as a result of which ITZ loaded SLN was deployed for topical ocular delivery through melt-emulsion sonication and low temperature-solidification technique. Many studies are being conducted in the world today concerning the improvement of therapeutic methods for corneal fungal infections due to the poor bioavailability of antifungal drugs arising from various protective mechanisms of eyes. One of the preferred mechanisms in advancing drugs for better penetration in the eye is the use of these nanosystems. The researchers reported the antimicrobial efficacy of these formulations by investigating *Aspergillus flavus* (Mohanty et al., 2015[72]). Other important triazoles are voriconazole (VRZ) that has been used in the treatment of candidiasis and aspergillosis infections in skin together with keratitis following topical administration (Marangon et al., 2004[69]; Al-Badriyeh et al., 2010[6]). Studies have suggested that nano-based VRZ formulation can be used to enhance VRZ permeation (Nassiri-Kashani et al., 2016[80]). One of those studies was the paper of Kumar and Sinha (2016[60]) that comprehensively examined SLNs for improved ocular delivery of VRZ, with satisfactory results obtained both *in vitro* and *in vivo.* They reported enhanced corneal drug permeation as well as revealing the non-irritating property of SLN compared to the pure drug (Kumar and Sinha, 2016[60]). Another study on VRZ-SLN was performed by Füredi and his colleagues in 2017 to develop a novel formulation of the ophthalmic dosage form. They worked on *Candida glabrata* and *Aspergillus flavus* in this study and finally reported that antifungal study using this formulation could inhibit the reproduction of fungus (Füredi et al., 2017[44]). Another commonly used triazole in either treatment or prophylaxis is fluconazole (FLZ), the focus of Gupta and colleagues for investigation in 2011 as a result of which FLZ-loaded SLNs were prepared by the aqueous diffusion method against cutaneous candidiasis which yielded satisfactory outcomes when compared with the pure drug (Gupta et al., 2013[47]). EL-Housiny et al. (2018[35]) generated FLZ-loaded SLNs topical gel to treat Pityriasis versicolor (the most common cutaneous dermatologic conditions worldwide) and observed impressive outcomes than candistan (clotrimazole) cream. The researchers used modified high shear homogenization and ultrasonication method to prepare SLN loaded with FLZ and then used Carbopol (CP 934) as a gelling agent to prepare FLZ-SLN topical gel. It has been reported that one of the advantages of using FLZ -loaded SLNs topical gel is that the treatment of this fungal infection via pure FLZ is feasible solely through oral therapy; thus, the topical application of this drug formulation can produce better results such as improved skin penetration due to enhanced contact between FLZ and skin. The researchers performed a clinical study of over 30 patients with Pityriasis versicolor to evaluate FLZ-SLNs topical gels whose findings were contrasted against Candistan cream. In this study, each patient was followed up by a clinical and mycological examination every week during therapy to assess the clinical efficacy and safety of different treatment regimens. The researchers eventually reported that the formulation appeared to be faster and more effective than topical Candistane (El-Housiny et al., 2018[35]). All of these studies on various azoles have indicated that the use of SLNs as an antifungal drug delivery system, especially for topical medicinal applications, has several important merits including good skin penetration, reduced side effects, biocompatibility, and enhanced therapeutic efficacy.

Another SLN study was carried out on GF, a drug frequently used to treat dermatophytosis. However, today, considering the several shortcomings of this drug such as its poor oral bioavailability and some other side effects, the topical application of drug delivery system of GF has been proposed. For this purpose, Anurak and his colleagues in 2011 developed a GF-loaded SLN by microemulsion method. The researchers showed that using this formulation makes GF release profiles be a delayed-release and also makes large scale production of SLNs quite viable (Anurak et al., 2011[10]).

### Nanostructured lipid carrier

Another drug delivery system is nanostructured lipid carriers (NLCs), being composed of both solid and liquid lipids as a core matrix (Aghebati-Maleki et al., 2020[1]). The utilization of these NPs in combination with antifungals yielded increased solubility. The diameter of NLCs ranges from 10 to 1000 nm so their minute particle size affects their solubility, biocompatibility, and rate of drug release. In addition, the shape of NLCs betters encapsulation efficiency, affects cellular uptake and receptor binding. NLCs as drug carriers improve permeability and bioavailability and lead to enhanced storage stability and prolonged half-life. Eventually, using NLCs as drug carriers protect the drugs from degradation in the body (Fang et al., 2013[37]; Haider et al., 2020[49]). In 2017, Fu et al. investigated the efficacy of AmB loaded, chitosan-modified, nanostructured lipid carriers (AmB-CH-NLC) for the effective ocular delivery of AmB for fungal keratitis using solvent emulsification and low temperature-solidification technology. It was reported that due to the cationic and mucoadhesive properties of CH, AmB-CH-NLC obtains better corneal penetration capacity over pure drugs. Based on research on rabbits' eyes, it was confirmed that AmB-CH-NLC could successfully penetrate the cornea (Fu et al., 2017[43]). On the basis of the findings of this study, the AmB-CH-NLC system may also be suggested as a treatment for fungal keratitis in the future given the difficulty of choosing the right drug for keratitis today.

### Cubic liquid crystalline nanoparticle (CUBOSOMES)

Cubosomes are extremely firm NPs formed from the lipid cubic phase and stabilized by a polymer-based outer corona (Barriga et al., 2019[15]). The cubosomes as drug carriers have a small size, low viscosity, large interfacial areas, and a hydrophobic core. The cavern-like structure of these NPs lead to efficient drug loading (Yang et al., 2012[122]; Zhang et al., 2020[124]). These NPs have properties such as high biocompatibility and bioadhesive that led Yang and his colleagues in 2014 to prepare AmB which is loaded in glyceryl monoolein (GMO) cubosomes to hone the oral delivery of AmB. They used rats in this *in vivo* study and reported that using these NPs could facilitate the oral delivery of AmB (Xu et al., 2014).

### Silver nanoparticle

Various studies on silver-containing compounds have shown that silver is a broad-spectrum antimicrobial agent, containing disruptive microbial cell membrane as well as an interfering power with DNA replication (Bhabra et al., 2009[16]; Vazquez-Muñoz et al., 2014[118]). Silver NPs (AgNPs) also have unique physical and chemical properties such as electrical, thermal, high electrical conductivity, and biological properties that lead these NPs to be widely used in medicine (Nozari et al., 2012[82]; Zhang et al., 2016[125]). Ahmad's study in 2016 involved *Candida albicans *and *Candida tropicalis* using AmB-conjugated biogenic silver NPs (AmB-bAgNPs). What they found in this study was that AmB-bAgNPs have a significant effect on enhancing their antifungal activities against *Candida* spp. compared to pure AmB. It was also reported that the antifungal stability of AmB-bAgNPs remained higher than the pure AmB for several days and this formulation could reduce the therapeutic cost and dose of AmB as well as its toxicity (Ahmad et al., 2016[2]). Another study in this area belongs to Tutaj et al., (2016[116]) who worked on hybrid-AmB silver NPs (AmB-Ag NPs). By investigating the antifungal activity of these NPs in *Aspergillus niger*, *Candida albicans* and *Fusarium culmorum* strains compared to Fungizone (AmB), the researchers reported that the formulation had a higher antifungal trait. They, therefore, suggested that using these NPs could open up a new avenue in the treatment of pathogenic fungi responsible for severe mycotic infections (Tutaj et al., 2016[116]). An additional research in this area was that of Hussain et al. (2019[55]) who investigated the enhanced antifungal efficacy of nystatin (NYS) and FLZ after conjugation with Ag; to this end, they designed NYS- and FLZ-coated Ag. The researchers employed strains of *Candida albicans* ATCC 10231 and *Aspergillus brasiliensis* ATCC 16404 using the agar tube dilution method to perform *in vitro* studies. What they discovered was that the combination of Ag with NYS and FLZ may have clinical implications in the treatment of fungal infections after observing the enhanced antifungal effects of NYS-Ag and FLZ-Ag (Hussain et al., 2019[55]). Mussin 's study in 2019 visualized the interaction between AgNP- *Malassezia furfur* and evaluated the synergism with KTZ to produce an antimicrobial gel based on carbopol formulated with AgNP-KTZ. Investigating the antifungal activity of this gel allowed the improvement of the topical therapy of superficial mycoses, reduction in the number of applications, and prevention of relapse (Mussin et al., 2019[78]). Thakur's study in 2019 indicated that the zinc ferrite nanoparticles and silver nanowires (ZnFe_2_O_4_@AgNWs) manifest both biofilm inhibition capacity for *Candida albicans* cells and potentially novel antifungal activities. They showed that AgNWs penetrated the biofilm matrix and transported ZnFe_2_O_4_ NPs into it, so ZnFe_2_O_4_ NPs could damage *Candida* cells by generating reactive oxygen species (ROS) (Thakur et al., 2019[112]). Szerencsés showed the same results by using citrate-coated AgNPs to inhibit the biofilm formation of Candida (Szerencsés et al., 2020[109]).

### Poly-lactic-co-glycolic acid nanoparticle

Many studies have suggested the use of PLGA NPs in the drug delivery system to optimize the therapeutic efficacy of many classical drugs and their toxicity as a result of the decreased dose of the drug. Several noteworthy benefits of PLGA NPs include biocompatibility, drug compatibility, and mechanical properties; besides, its hydrolysis results in the formation of monomers of lactic acid and glycolic acid metabolites. Since these two monomers are androgens and are easily metabolized by body through the Krebs cycle or TCA cycle, PLGA is associated with poor systemic toxicity. PLGA NPs protect the drug against degradation and improve its stability (Yang, 2019[121]). Among these studies, a study by Amaral et al. (2010[8]) can be pointed out, in which immunoprotective peptide P10 (the major diagnostic antigen secreted by *Paracoccidioides brasiliensis*) loaded on PLGA was utilized to treat paracoccidioidomycosis. Male BALB/c mice infected with *Paracoccidioides brasiliensis* (inoculated intratracheally) were used in this study. They also deployed animal models for which a combination of sulfamethoxazole/trimethoprim with the P10 peptide entrapped within PLGA was utilized and observed fulfilling results in some treatments due to P10 elicits, a Th1-like immune response which can control fungal infection (Amaral et al., 2010[8]). In 2014, Liu and his colleagues managed to prepare AmB-loaded diblock copolymer D-α-tocopheryl polyethylene glycol 1000 succinate-b-poly (ε-caprolactone-ran-glycolide) (PLGA-TPGS) by a modified nanoprecipitation method. To investigate the effect of these NPs, they first performed antifungal activity *in vitro* on a strain of *Candida albicans* using a paper-plate technique and reported that AmB-NPs have an antifungal activity similar to that of free AmB. The *in vivo* therapeutic efficacy of AmB-NPs was then tested using an animal model of BALB/c mice which showed high stability of AmB-NPs in this study compared to free AmB (Liu et al., 2014[64]). Other studies include Das' study on pulmonary delivery of VRZ as a result of which PLGA NPs containing antifungal drug VRZ was developed. The *in vivo* investigations indicated that the accumulation of VRZ-loaded PLGA in lungs was significantly higher than that of a pure drug; in addition to this, the prolonged retention of particles in lungs was observed. They finally showed that VRZ-loaded PLGA is a proper system for delivering a drug in deep lung tissue in high concentrations for a prolonged period (Das et al., 2015[32]). Working on AmB entrapped within PLGA and incorporated with dimercaptosuccinic acid (NANO-D-AMB), affirmed by the in vitro and *in vivo* findings, helped confirm that NANO-D-AMB improves AmB delivery (Souza et al., 2015[105]). Radwan et al. (2017[91]) prepared AmB loaded to PEGylated polylactic-polyglycolic acid copolymer (PLGA-PEG) NPs and observed that the MIC of AmB loaded to PLGA-PEG NPs against *Candida albicans* was reduced compared with fungizone (free AmB). On the other hand, studies on rats disclosed that the oral delivery system of the NPs manifested minimal toxicity and better efficacy compared to fungizone (Radwan et al., 2017[91]). In another study, Ahmed and Aljaeid (2017[3]) developed optimized ketoconazole (KTZ) PLGA NPs with *in situ* gel (ISG) formulation for ophthalmic drug delivery in an attempt to streamline ocular KTZ delivery. Using this formulation, the researchers observed the enhanced antifungal activity against the standard strain of *Candida albicans* ATCC 76615 and also reported that these NPs could be used in the treatment of both keratitis and endophthalmitis owing to their ability for transcorneal permeation (Ahmed and Aljaeid, 2017[3]).

### Gelatin nanoparticle

Gelatin (a natural polymer derived from collagen) is one of the substances used in the production of NPs. Several noticeable advantages of gelatin as NPs are their biodegradable, non-toxic, non-immunogenic biocompatible nature with human tissues as well as their easy crosslinking and potential to modify chemically. Gelatin NPs are delivered to special tissues without compromising drug stability and concentration which is considered a plus point (Singh and Mishra, 2014[101]; Sabet et al., 2017[96]). In a study by Ahsan and Rao in 2017 on controlling infection and inflammation in keratitis, they prepared double-desolvation KTZ loaded gelatin nanostructures. In their study on rats' corneas infected with *Aspergillus flavus*, the researchers found that these NPs can enhance drug residence time, down-regulate inflammation, and release antifungal drugs (Ahsan and Rao, 2017[4]).

### Alginate nanoparticle 

Alginic acids or alginates are natural polymers consisting of linear copolymers and varying amounts of (1→4′)-linked β-d-mannuronic acid and α-l-guluronic acid residues (Rehm and Valla, 1997[92]). Alginate is often obtained from brown algae and certain bacteria and is used for drug delivery applications nowadays (Spadari et al., 2017[108]). Alginate is a natural, non-toxic, biocompatible, biodegradable, water-soluble, and non-immunogenic polymer. One of the noticeable benefits of alginate as NPs is the sustained release of drugs so that they can be constantly released into the tissue. The different drug release mechanisms of these NPs are diffusion and matrix erosion for water-soluble drugs and poorly water-soluble drugs, respectively (Spadari et al., 2019[107]). In this regard, Pandey and his colleagues worked on two CLT and ECN drugs in 2005 and managed to produce cation-induced controlled gelification of alginate, CLT-loaded alginate nanoparticles, and ECN-loaded alginate nanoparticles. These two NPs were orally administered to mice and thus improved the oral bioavailability of the drugs. Other benefits of this formulation are their simplicity of preparation and increased duration of sustained drug release (Pandey et al., 2005[85]). Sangeetha et al. (2007[99]) scrutinized systemic candidiasis both *in vitro *and* in vivo *(the* in vivo *study was carried out on candidiasis induced mice models) conditions. Sodium alginate nanospheres of AmB were prepared by the controlled gellification method in this study. Having compared the nanosphere-bound drug with the free drug, the researchers reported that upon using this formulation, a significant reduction in colony-forming units (CFU) in the liver and lungs was observed. This finding, in turn, contributes to a reduction in the total dose required for therapy and, consequently, the degree of drug toxicity (Sangeetha et al., 2007[99]).

### Magnetic nanoparticle

Magnetic NPs (MNPs) (such as iron oxide NPs) are a class of NPs that can be manipulated using magnetic fields and due to magnetic properties, antibiotics included in such complexes can be delivered to specific areas using a magnetic field (Akbarzadeh et al., 2012[5]; Niemirowicz et al., 2016[81]). Magnetic nanoparticles possess multimodal targeting potential and have thermal therapy applications due to their ability to convert energy from an alternating magnetic field to thermal energy (Hauser et al., 2015[51]). In 2016, Niemirowicz and his colleagues studied two polyene antifungals, AmB and NYS, using Massart's procedure; namely, MNP-AmB and MNP-NYS. After comparing MNP with pure drugs on *Candida* strains, they reported that these formulations were able to prevent *Candida* biofilm formation and improve their biocompatibility (Niemirowicz et al., 2016[81]). Muñoz-Escobar and Reyes-López investigated the antifungal activity of polycaprolactone-copper fibers (PCL-CuONPs) in *Candida albicans*, *Candida glabrata*, and *Candida tropicalis *in 2020. They showed that the formation of mycelia was inhibited in the presence of synthesized NPs, a pre-requisite in the dimorphic transition from yeast to mycelial form in superficial fungal infections caused by *Candida *(Muñoz-Escobar and Reyes-López 2020[76]).

### Chitin nanoparticle

Other NPs used today are chitin nanogels (CNGs) which are natural polymeric NPs with great potential in the field of nanotherapeutics. Chitin is an excellent material for the synthesis of nanoparticles as a natural renewable resource, demonstrating biodegradable, biocompatible, and non-toxicity traits (Zhang et al., 2018[123]; López et al., 2020[65]). Mohammed and colleagues used FLZ-loaded chitin nanogels (FLZ-CNGs) in the treatment of corneal fungal infections in 2013 to overcome barriers such as minimal dose absorption due to unique anatomy and physiology of eyes. By producing these NPs, they managed to detect the good antifungal activity of this formulation in comparison to the pure drug against *Candida tropicalis*, which has a determining role in higher uptake by fungal cells. The researchers used the cornea obtained from the porcine eye to investigate *ex vivo* corneal permeation which ultimately demonstrated the effective penetration of FLZ-CNGs into the deeper sections of the cornea (Mohammed et al., 2013[71]).

### Chitosan nanoparticle

Chitosan obtained by chitin acetylation is suitable for the formation of nanogels. Chitosan is a non-toxic and biocompatible polymer with strong mucoadhesive properties, which is used now either as a matrix or coating for NPs (Onyebuchi and Kavaz, 2019[83]). In 2013, Song et al. succeeded in preparing AmB-loaded NPs based on poly (lactic acid)-grafted-chitosan (AmB/PLA-g-CS) for the ocular delivery of AmB by dialysis. *In vitro* antifungal activity was performed by researchers against *Candida albicans* using the M27-A3 (2008) guidelines of clinical and laboratory standards institute indicating that AmB-loaded NPs have an antifungal potential similar to that of the free drug. After investigating ocular pharmacokinetics, they reported the prolonged residence time on the ocular surface. On the other hand, in a corneal penetration study, they observed the penetration of NPs into the cornea (Song et al., 2013[103]). In another study, Vasquez-Marcano et al., in 2018 used AmB-loaded chitosan-coated poly (ε-caprolactone) NPs (AmB-loaded CS-coated PCL NPs) produced by the nanoprecipitation method for the oral delivery of AmB and a reduction in its toxicity. They also revealed that by using NPs, the antifungal activity against *Candida parapsilosis* strain intensifies as against the pure AmB. It was also divulged that these NPs could safely release AmB via the oral route (Vásquez Marcano et al., 2018[117]). Paul and his colleagues also achieved similar results in 2018 by preparing Chitosan-coated poly lactic-co-glycolic acid NPs of VRZ (VChNP), affirming that using this formulation, compared with free VRZ, depicts effective pulmonary delivery and boosted bioavailability (Paul et al., 2018[86]). Sandhya et al. (2018[98]) also observed that by preparing AmB encapsulated sulfated CS NPs (AmpB-SCNPs), synthesized NPs manifested higher intracellular killing of *Candida glabrata* in infected RAW 264.7 macrophage cell lines. Studies have shown that both lectin and CS are both biocompatible and biodegradable polymers, which is why Lecithin/chitosan nanoparticles (L/CS NPs) are highly regarded in the drug delivery system these days. To this end, Chhonker et al. made use of AmB loaded L/CS NPs prepared by ionic gelation and achieved the prolonged release of AmB with a reduction in the dosage of pure drug and the consequent side effects. In the *in vitro *study of *Candida albicans* and *Aspergillus fumigatus*, the researchers perceived the potential antifungal efficacy by preparing AmB loaded L/CS NPs and similarly, based on the *in vivo* study conducted in New Zealand on albino rabbit eyes, improved bioavailability compared to the free drug was noted. Thus, they reported that this formulation holds a higher ocular tolerance than the marketed formulation, however, further studies are necessary for commercial applications (Chhonker et al., 2015[27]). Khan and his colleagues (2020) synthesized Methylglyoxal Chitosan Nanoparticles (MGCN) against fluconazole-resistant *Candida albicans* and investigated them both *in vitro* and in a mouse model. Synthesized NPs not only evinced higher intracellular killing of *Candida albicans* in infected macrophages of female BALB/C mice, but also inhibited yeast to hyphae transition, and enhanced *in vivo* efficacy and reduced toxicity (Khan et al., 2020[58]).

### Polycaprolactone nanoparticle

Polycaprolactone (PCL) is one of the synthetic biodegradable polymers for drug delivery and biomedical applications. PCL NPs depict low particle size and high flexibility. PCL-based NP formulations have slow release due to high crystalline nature. Also, these NPs have less water permeability and consequently a very slow degradation rate (Ashour et al., 2019[11]; Muñoz-Escobar and Reyes-López, 2020[76]). Chandasana et al. investigated natamycin for the treatment of mycotic keratitis (natamycin 5 %, FDA approved and commercially available for topical ocular use) and benefited from the formulation of natamycin encapsulated poly-D-glucosamine functionalized polycaprolactone NPs (PDG-PCL NPs) and finally succeeded in devising a targeted corneal nanoformulation to reduce the dose and dosing frequency of natamycin. Using this method hand in hand with the *in vitro* studies, the prolonged release of natamycin was reported and subsequently, the *in vivo* conditions yielded highly satisfactory outcomes as well (Chandasana et al., 2014[25]). In the same vein, Lucena et al. (2018[66]) also achieved impressive results by preparing ITZ loaded PCL nanocapsules (ITZ-NC) for the treatment of vulvovaginal candidiasis after topical application in the vagina. Having conducted the *in vivo* study of female BALB/c mice, the researchers observed the effect of ITZ-NC formulation on reducing *Candida albicans* and the arising inflammation. Based on their studies, it was reported that their nanocapsules have the potential to increase drug contact with the mucosa and open new perspectives for the treatment of this disease (Lucena et al., 2018[66]). All studies are summarized in Table 1(Tab. 1) (References in Table 1: Ahmad et al., 2016[2]; Ahmed and Aljaeid, 2017[3]; Ahsan and Rao, 2017[4]; Aljaeid and Hosny, 2016[7]; Amaral et al., 2010[8]; Anurak et al., 2011[10]; Bhalekar et al., 2009[17]; Butani et al., 2016[22]; Cassano et al., 2016[24]; Chandasana et al., 2014[25]; Chhonker et al., 2015[27]; Das et al., 2015[32]; El-Housiny et al., 2018[35]; Fu et al., 2017[43]; Füredi et al., 2017[44]; Gupta et al., 2013[47]; Hussain et al., 2019[55]; Jain et al., 2010[56]; Kenechukwu et al., 2017[57]; Khan et al., 2020[58]; Kumar and Sinha, 2016[60]; Liu et al., 2014[64]; Lucena et al., 2018[66]; Mohammed et al., 2013[71]; Mohanty et al., 2015[72]; Muñoz-Escobar and Reyes-López 2020[76]; Mussin et al., 2019[78]; Niemirowicz et al., 2016[81]; Pandey et al., 2005[85]; Paul et al., 2018[86]; Radwan et al., 2017[91]; Sandhya et al., 2018[98]; Sangeetha et al., 2007[99]; Sanna et al., 2007[100]; Song et al., 2013[103]; Souto et al., 2004[104]; Souza et al., 2015[105]; Szerencsés et al., 2020[109]; Thakur et al., 2019[112]; Tutaj et al., 2016[116]; Vásquez Marcano et al., 2018[117]; Xu et al., 2014[120]).

## Toxicity of Nanoparticles

NPs are being increasingly employed in a variety of sectors. NPs have been studied for cell toxicity, immunotoxicity, and genotoxicity. Tetrazolium-based assays such as MTT, MTS, and WST-1 are used to determine cell viability. Cell inflammatory response induced by NPs is checked by measuring inflammatory biomarkers, such as IL-8, IL-6, and tumor necrosis factor, using ELISA. Lactate dehydrogenase assay is used for cell membrane integrity (Bahadar et al., 2016[13]). Different types of cell cultures, including cancer cell lines have been employed as *in vitro* toxicity models. It has been generally agreed that NPs interfere with either assay materials or detection systems. Based on multiple applications in multiple areas, human exposure to NPs, both intentionally and unintentionally, is inevitable. Before being considered for human application, all nanoproducts are subjected to toxicological studies and for this purpose, several experimental studies have been carried out. To meet this regulatory requirement, some toxic effects of nanomaterials have been evaluated, but according to reports, the toxicological data derived so far are conflicting and inconsistent (Badman et al., 2020[12]). Toxicological studies provide a sound grounding for the protection of both humans and environment. Therefore, on the basis of available experimental models, it may be inaccurate to list some of the more valuable NPs as being toxic to biological systems and vice versa. Considering the potential applications of NPs in many fields and to address the knowledge gap, the relevant toxic effects of NPs should be assessed by utilizing internationally agreed and unbiased *in vivo* toxicological models, targeting the vital systems (Pacheco-Blandino et al., 2012[84]). However, we are of the opinion that designing, adapting, and validating such new models in the future for toxicity testing, route of exposure, coating material and sterility of NPs, and type of cell cultures need to be carefully examined (Crisponi et al., 2017[29]). Moreover, the US FDA as a public health agency has also recently taken the important issue of toxic effects associated with products containing NPs into account and does not consider them either totally safe or harmful for human use, maintaining that each product must be subjected to regulation (Bahadar et al., 2016[13]).

## Conclusion

Today, fungal infections gain ever-increasing significance given the growing annual incidences and mortality rate emanating from such infections. Although today the antifungals which are effective in treating these fungal infections are commercially bountiful, researchers have sought ways to optimize the existing drugs thanks to limited penetration through tissue, poor aqueous solubility, reduced bioavailability, drug efficacy and drug resistance, side effects, and poor drug pharmacokinetics. Hence, during the last two decades, the use of NPs drug-delivery systems to enhance the scope of antifungal functions has gained a prominent place in antifungals' optimization. The main merits of these NPs are their small size and large surface area that make them ideal candidates for various applications and able to overcome the limitations of the existing drugs, though partially. Yet, considering all the studies conducted in this field, except the few cases mentioned (such as liposomal AmB, marketed in nanoformulations), none of these formulations are commercially available largely owing to preclinical problems concerning the studies and clinical trials as well as the limitations surrounding the utilization of these NPs.

## Acknowledgements

The study was approved by the ethics committee of Tabriz University of Medical Sciences (Grant no. IR.TBZMED.VCR.REC. 1398.103).

## Funding information

This work was supported financially by Research Vice-Chancellor, Tabriz University of Medical Sciences, Tabriz, Iran.

## Conflict of interest

The authors declare that they have no conflict of interest.

## Figures and Tables

**Table 1:**
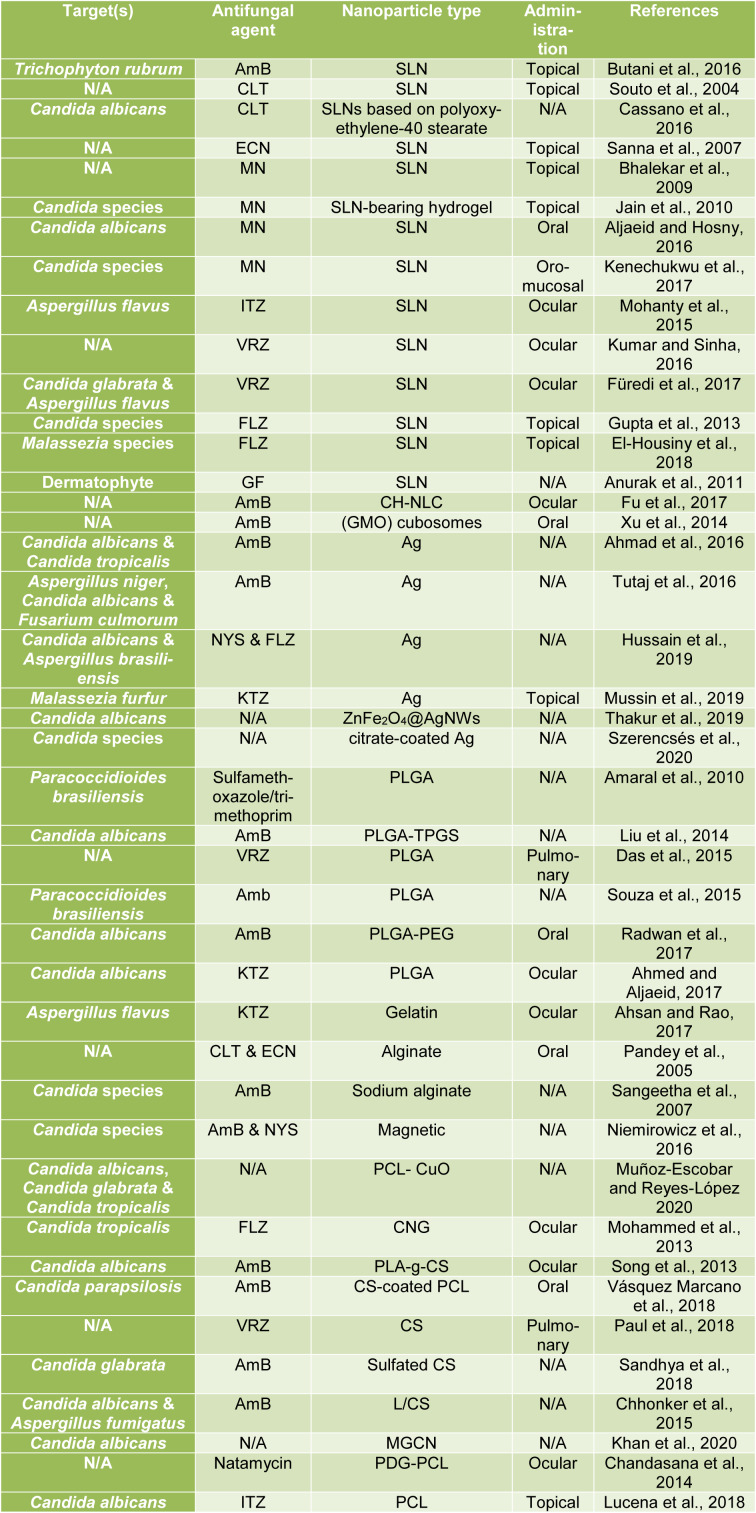
Studies conducted in nanoparticles for antifungal drugs delivery
